# Analysis of the Trends and Influencing Factors for Postoperative Cough in Patients with Esophageal Cancer Based on Patient-Reported Outcomes

**DOI:** 10.1245/s10434-024-15413-7

**Published:** 2024-05-18

**Authors:** Jie Sun, Rui Liang, Qing Zhang, Na Liu, Qingmiao Zhu, Qi Li, Dan Yu, Yuan Yu, Jianjun Qin

**Affiliations:** Department of Thoracic Surgery, National Cancer Center/National Clinical Research Center for Cancer/Cancer Hospital, Chinese Academy of Medical Sciences and Peking Union Medical College, Chaoyang District, Beijing, China

**Keywords:** Cough, Esophageal cancer, Patient-reported outcomes

## Abstract

**Background:**

Cough is a common symptom that affects patients’ recovery and quality of life after esophagectomy. This study sought to investigate trends in postoperative cough and the factors that influence cough.

**Methods:**

A total of 208 of 225 patients were enrolled in this study. The Mandarin Chinese version of the Leicester Cough Questionnaire was administered the day before surgery and at three time points (1 week, 1 month, and 3 months) after esophagectomy to assess patient-reported outcomes.

**Results:**

All patients’ LCQ-MC scores after surgery were lower than presurgery (*P* < 0.05), with the lowest score found 1 week after esophagectomy. Factors associated with a cough 1 week after surgery included clinical stage of cancer (OR 0.782, 95% CI 0.647–0.944, *P* = 0.011), anastomotic position (OR 1.241, 95% CI 1.069–1.441, *P* = 0.005), duration of surgery (OR 0.759, 95% CI 0.577–0.998, *P* = 0.049), and subcarinal lymph node dissection (OR 0.682, 95% CI 0.563–0.825, *P* < 0.001). Factors associated with a cough one month after surgery included clinical stage (OR 0.782, 95% CI 0.650–0.940, *P* = 0.009), anastomotic position (OR 1.293, 95% CI 1.113–1.503, *P* = 0.001), and maintaining a semi-reclining position (OR 1.440, 95% CI 1.175–1.766, *P* < 0.001). Factors associated with a cough 3 months after surgery were clinical stage (OR 0.741, 95% CI 0.591–0.928, *P* = 0.009) and anastomotic position (OR 1.220, 95% CI 1.037–1.435, *P* = 0.016).

**Conclusions:**

This study showed that the factors influencing postoperative cough differed over time following esophagectomy. These results may warrant prospective intervention to better manage patients undergoing surgery for esophageal cancer to prevent postoperative cough.

Esophageal cancer (EC) is commonly treated with a multimodality approach involving surgery, radiotherapy, and/or chemotherapy, depending on the stage and location of the tumor. Despite extensive changes in therapeutic modalities, surgery remains an essential component of the curative treatment of EC. Cough is one of the most common symptoms in patients with EC after surgery, and it can adversely affect the postoperative recovery and quality of life of these patients.^1^

Cough is a protective reflex of the respiratory tract.^2^ The act of coughing involves many systems in the body, including sensory neurons, afferent nerves, efferent nerves, and effector organs. A pathological change in any one of these systems may result in coughing.^3^ To date, few studies have reported the factors that influence postoperative coughing in patients with EC. Consequently, the factors associated with coughing after esophagectomy remain unclear.

Recently, patient-reported outcomes (PROs) have been suggested as a relevant indicator for assessing treatment. By using interviews, self-rating scales, and other data collection tools, PROs can directly acquire data reported by patients, thus providing a measurement approach for assessing the treatment effects in clinical practice.^4^ In our present study, we assessed postoperative cough in EC patients by using PROs derived from the Mandarin Chinese version of the Leicester Cough Questionnaire (LCQ-MC). The risk factors associated with postoperative cough also were investigated for reducing the occurrence of cough in EC patients after esophagectomy.

## Patients and Methods

### Study Design and Participants

A total of 225 consecutive patients with EC who underwent esophagectomy in the Thoracic Surgery Department at the Cancer Institute and Hospital of China between June 2021 and December 2021 were assessed for trial eligibility. Of these 225 patients, 208 patients who met the eligibility criteria were finally included in this study. The inclusion criteria were as follows: (1) patients diagnosed with esophageal cancer or esophagogastric junction cancer and treated with surgery; (2) age > 18 years; (3) ability to provide informed consent; (4) either patient or primary caregiver was conscious and could communicate; and (5) mild or no cough before surgery according to LCQ-MC (≤9, severe cough; >9 and ≤15, moderate cough; >15 and ≤21, mild or no cough). The exclusion criteria were as follows: (1) informed consent not obtained; (2) presence of other respiratory diseases (e.g., tuberculosis, asthma, and chronic obstructive pulmonary disease); (3) history of lung resection; and (4) history of severe complications post-surgery (e.g., pulmonary infection, respiratory failure, and anastomotic fistula).

Methods of surgery include the McKeown approach (cervical, right thoracic, and abdominal 3-incision surgery) for cervical anastomosis and the Ivor-Lewis approach (abdominal and right thoracic 2-incision surgery) for intrathoracic anastomosis. The options for the surgical approach included open and minimally invasive techniques. With a standard lymphadenectomy, we usually do two-field lymphadenectomy. The optimal surgical procedure for Siewert type II adenocarcinoma or squamous cell carcinoma around esophago-gastric junction was, however, not standardized because the optimal extent of lymph node dissection was unknown. The 208 study patients received standard drug treatment for proton pump inhibitor (PPI) after surgery.

Questionnaires were administered as PROs at four different timepoints: 1 day before esophagectomy; 1 week after surgery; 1 month after surgery; and 3 months after surgery. The clinical stage of the disease was determined by using the UICC TNM grading system (7th edition).

The study protocol was approved by the Cancer Foundation of China (Project Number: LC2020C02) and the CAMS Innovation Fund for Medical Sciences (CIFMS) (Project Number: 2021-I2M-C&T-B-069 and 2022-I2M-C&T-B-084). The research plan was approved by the Ethics Committee of the National Cancer Center before the study (Approval No. 22/271-3473). Each potential participant was informed about the purpose of the study, what his/her involvement would be, and the right to withdraw at any time without repercussions. A consent form was signed by all study participants.

## Measures

### The LCQ-MC

The LCQ-MC is a useful tool to assess the severity of cough and its impact on patients’ quality of life. It is more flexible than other cough-related questionnaires and has practical relevance in the clinical evaluation and diagnosis of cough.^5^ The LCQ-MC includes 19 multiple choice questions, with seven scores for each question; these questions are related to physical, psychological, and social dimensions, including eight physiological items, seven psychological items, and four social items. A higher score indicates a milder cough. The score for each dimension is the average of the scores of all items in that dimension. The total score is the sum of the average scores of all three dimensions. The LCQ scale is highly effective for evaluating chronic and acute cough, can be easily completed, and can be self-administered in less than 5 min. All the enrolled patients reported their results through a WeChat mini program based on the LCQ-MC.

## Statistical Analysis

Data analysis was performed by using SPSS 25.0 software. Quantitative data were expressed as frequency and percentage (%). A standard chi-square test was used to compare distinct groups. Normally distributed data were analyzed using an independent sample t-test and presented as mean ± standard deviation. Nonnormally distributed data were analyzed using the Wilcoxon rank-sum test and presented as median and 25th (Q1) and 75th (Q3) quartiles . Multivariate logistic regression analysis was conducted to determine the independent risk factors for cough. *P* < 0.05 was considered statistically significant.

## Results

### Clinical and Pathological Data of the Patients

**Figure 1:**
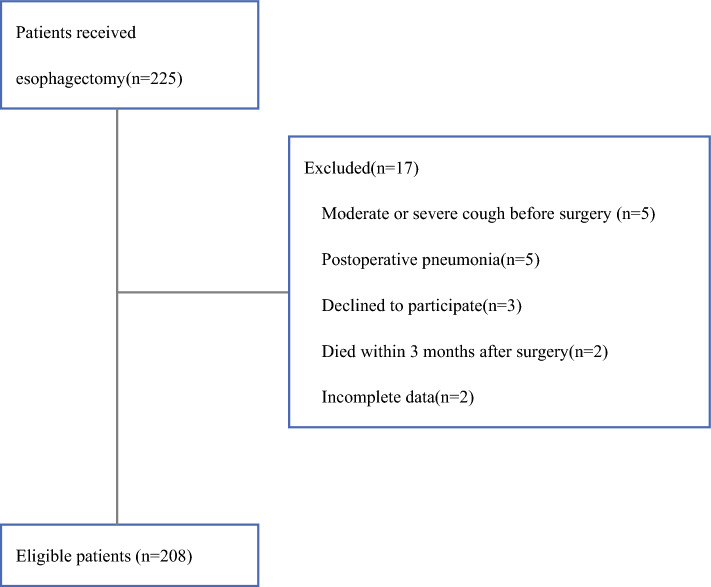
Study flow chart

Seventeen of 225 patients considered for study participation were excluded, as shown in Fig. 1. The remaining 208 patients were enrolled in the study, and their demographic and clinical characteristics are shown in Table 1. Ninety-four patients with smoking history received smoking cessation education and guidance after admission, and none of these patients showed relapse within 3 months after they quit smoking.

**Table 1:** Clinical characteristics and univariate analysis of postoperative cough in patients after esophagectomy one week, one month and three months after surgery

Item		Entire study population	One week after surgery		One month after surgery			Three months after surgery		
		(*n* = 208)	LCQ-MC	*P*	LCQ-MC		*P*	LCQ-MC		*P*
Gender	Male	178	9.8±2.1	0.878	12.7±2.5		0.56	15.6±1.9		0.654
	Female	30	9.8±1.7		12.7±2.0			15.9±1.9		
Age, years	< 60	64	10.9±2.0	<0.001*	13.8±2.0		<0.001*	16.7±1.6		<0.001*
	≥ 60	144	9.3±1.9		12.3±2.0			15.4±1.9		
Neoadjuvant therapy	Neoadjuvant therapy	174	9.5±1.8	0.617	12.7±1.7		0.862	15.7±1.3		0.847
	No neoadjuvant therapy	34	10.1±2.0		13.1±2.2			15.8±2.0		
History of smoking	Smoking	94	9.5±1.9	0.156	12.5±2.0		0.212	15.6±1.9		0.303
	No smoking	114	9.9±2.1		12.8±2.2			15.9±1.8		
Lesion location	Proximal third	18	10.6±1.8	0.183	13.5±2.3		0.27	16.6±1.5		0.17
	Middle third	75	9.8±2.1		12.5±2.1			15.5±1.9		
	Distal third	104	9.5±1.9		12.7±2.0			15.8±1.9		
	Esophagogastric junction	11	9.2±1.9		12.1±1.7			15.3±1.4		
Surgical approach	Open	22	9.1±2.1	0.136	12.9±2.1		0.577	15.5±2.1		0.574
	Minimally invasive	186	9.8±2.0		12.7±2.1			15.8±1.88		
Tumor histology	Squamous cell carcinoma	144	9.5±2.6	0.433	12.6±2.1		0.589	15.7±1.9		0.693
	Adenocarcinoma	59	9.6±1.9		12.9±2.1			15.9±1.7		
	Others	5	8.7±2.6		12.0±1.7			15.2±2.7		
Clinical stage	I	40	10.9±1.9	<0.001*	13.8±2.0		<0.001*	16.8±1.6		<0.001*
	II	89	9.9±2.0		12.8±2.0			15.9±1.7		
	III	73	9.0±1.7		12.1±2.0			15.0±1.9		
	IV	6	7.7±1.0		11.2±1.3			14.8±1.0		
anastomotic position	Cervical anastomosis	142	9.5±1.9	0.011*	12.4±1.9		0.002*	15.6±1.9		0.015*
	intrathoracic anastomosis	66	10.4±2.2		13.5±2.4			16.2±1.9		
Cervical lymph node dissection	Yes	60	9.6±1.8	0.528	12.5±1.7		0.675	15.8±1.7		0.541
	No	148	9.7±2.1		12.7±2.2			15.7±1.9		
Recurrent laryngeal nerve lymph node dissection	Yes	167	9.8±2.0	0.206	12.7±2.1		0.71	15.7±1.9		0.95
	No	41	9.4±2.0		12.8±1.9			15.7±1.9		
Subcarinal lymph node dissection	Yes	172	9.5±2.0	<0.001*	12.6±2.1		0.27	15.7±1.9		0.815
	No	36	11.0±1.6		13.0±2.0			15.8±1.		
Duration of surgery, hours	< 3	16	11.3±2.1	<0.001*	13.6±2.2		0.167	16.0±2.2		0.437
	3–4	69	10.2±2.2		12.3±1.9			15.4±1.6		
	4–5	96	9.5±1.7		12.8±2.1			15.9±1.9		
	> 5	27	8.3±1.7		12.2±2.7			16.0±2.4		
First time of eating after operation, days	> 7	82	9.7±1.8	0.642	12.7±1.9		0.486	15.0±1.9		0.56
	7–14	46	9.5±2.1		12.3±2.1			15.5±1.		
	14–30	56	9.9±2.2		12.9±2.1			15.9±1.8		
	> 30	24	10.1±2.0		12.9±2.5			16.1±1.8		
Chest tube duration, days	≤ 7	67	9.5±1.9	0.399	12.5±2.0		0.657	15.6±1.9		0.629
	> 7	141	9.8±2.1		12.8±2.1			15.8±1.8		
Chest tube size	28F	37	8.6±2.0	<0.001	12.6±2.1		0.9	15.7±1.9		0.8
	16F	90	9.7±1.7		12.8±1.9			15.8±1.9		
	14F	81	10.3±2.1		12.8±2.2			15.9±1.8		
Maintaining a semi-reclining position	Very little time	10	8.7±1.3	0.283	37	11.8±2.2	<0.001*	29	15.1±2.6	0.43
	Now and then	57	9.6±2.2		70	12.4±2.1		72	15.4±2.2	
	Frequently	141	9.7±1.8		101	13.2±1.9		107	15.7±2.0	
Gastric tube duration, days	None	72	9.8±1.9	0.473	12.9±1.9		0.153	15.8±1.9		0.66
	≤7	7	8.7±2.6		11.0±2.0			14.9±2.5		
	7-14	14	9.5±1.2		11.0±2.0			15.7±1.4		
	> 14	115	9.8±2.1		12.72±2.2			15.7±1.8		

### LCQ-MC Score Trend and Distribution Before and After Surgery

**Figure 2:**
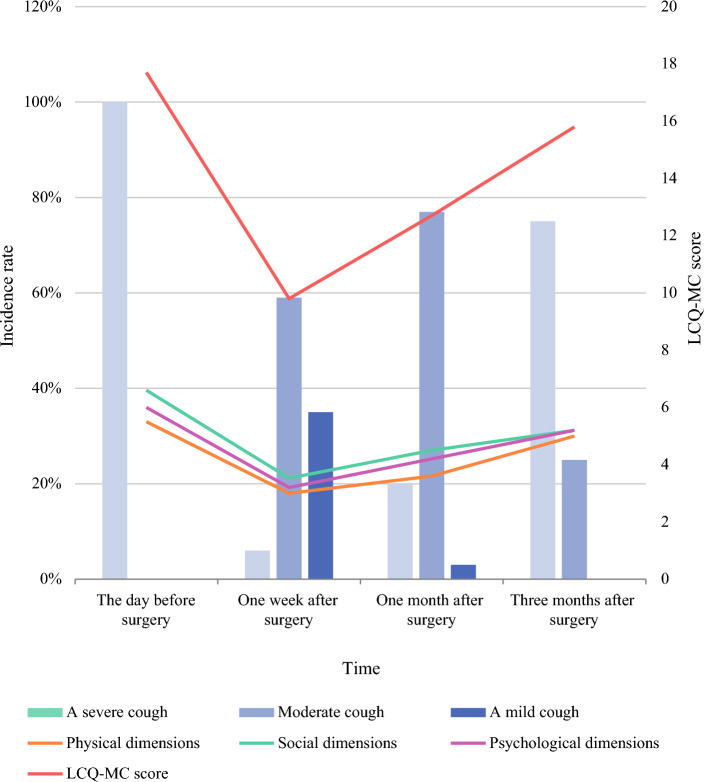
Trends in the total LCQ-MC score and scores of the different dimensions pre-and post-surgery (line chart) and The proportion of patients reporting mild, moderate or severe cough using the LCQ-MC at different times (bar chart)

A significant difference (*P* < 0.05) was noted in the LCQ-MC scores of all four timepoints. After surgery, all the LCQ-MC scores, including the total scores and scores for each dimension, were lower (indicating more severe cough) than those before surgery (Fig. 2).

Figure 2 shows the LCQ-MC scores as percentages at the four timepoints. On the LCQ-MC scale, the proportion of patients reporting mild or no cough decreased from 100% before surgery to 6% at 1 week after surgery, 20% at 1 month after surgery, and 75% at 3 months after surgery. In contrast, the proportion of patients who reported moderate and severe cough first increased and then decreased after surgery.

### Univariate Analysis of Post-surgery Cough

Univariate analyses revealed that the factors associated with cough at 1 week after surgery were age, clinical stage, anastomotic position, subcarinal lymph node dissection, duration of surgery, chest tube size, and maintaining a semi-reclining position. At 1 month after surgery, the factors associated with cough were age, clinical stage, anastomotic position, and maintaining a semi-reclining position. The factors associated with postoperative cough at 3 months after surgery were age, clinical stage, and anastomotic position (Table 1).

### Multiple Logistic Regression Analysis of Postoperative Cough

The risk factors found to be significantly associated with cough in the univariate analyses were then subjected to multivariate logistic regression. The results showed that, at 1 week after surgery, clinical stage (odds ratio [OR] 0.782, 95% confidence interval [CI] 0.647–0.944, *P* = 0.011), duration of surgery (OR 0.759, 95% CI 0.577–0.998, *P* = 0.049), and subcarinal lymph node dissection (OR 0.682, 95% CI 0.563–0.825, *P* < 0.001) were negatively associated with cough, whereas anastomotic position (OR 1.241, 95% CI 1.069–1.441, *P* = 0.005) was positively associated with cough. At 1 month after surgery, clinical stage (OR 0.782, 95% CI 0.650–0.940, *P* = 0.009) was negatively associated with cough, whereas anastomotic position (OR 1.293, 95% CI 1.113–1.503, *P* = 0.001) and maintaining a semi-reclining position (OR 1.440, 95% CI 1.175–1.766, *P* < 0.001) were positively associated with cough. At 3 months after surgery, clinical stage (OR 0.741, 95% CI 0.591–0.928, *P* = 0.009) was negatively associated with cough, whereas anastomotic position (OR 1.220, 95% CI 1.037–1.435, *P* = 0.016) was positively associated with cough (Table 2).

**Table 2:** Multivariate regression analysis of postoperative cough

Stage	Variables	Wald	*B*	Odds ratio	95% CIs	*p* value
One week after surgery	Clinical stage	6.515	− 0.246	0.782	0.647–0.944	0.011
	Anastomotic position	8.018	0.216	1.241	1.069–1.441	0.005
	Duration of surgery (hours)	3.886	− 0.275	0.759	0.577–0.998	0.049
	Subcarinal lymph node dissection	15.54	− 0.38	0.682	0.563–0.825	< 0.001
One month after surgery	Clinical stage	6.787	− 0.246	0.782	0.650–0.94	0.009
	Anastomotic position	11.259	0.257	1.293	1.113–1.503	0.001
	Maintaining a semi-reclining position	12.295	0.365	1.44	1.175–1.766	< 0.001
Three months after surgery	Clinical stage	6.803	− 0.3	0.741	0.591–0.928	0.009
	Anastomotic position	5.766	0.199	1.22	1.037– 1.435	0.016

## Discussion

Our previous study showed that patients experienced a variety of symptoms after esophagectomy, the most common of which were cough, hoarseness, and reflux heartburn.^6^ Chronic cough may persist for a long time and affect patients’ postoperative rehabilitation, thus increasing their psychological burden and adversely affecting their physical and mental health.^7,8^ In the present prospective study, the LCQ-MC was used to assess postoperative cough in patients with EC. The results showed that postoperative cough after esophagectomy first decreased and then increased; however, the level of postoperative cough at 3 months after surgery was still lower than the preoperative level. Moreover, the extent of postoperative cough was influenced by different factors at different periods within the first 3 months after surgery.

Clinical stage and anastomotic position were significantly associated with postoperative cough at 3 months post-surgery. Patients with cervical anastomosis had lower LCQ-MC scores than those with intrathoracic anastomosis. This finding was likely related to reflux, as Park et al. and Kim et al. reported that cervical anastomosis was a significantly higher risk factor for reflux and aspiration pneumonia than thoracic anastomosis. Kim et al. also found that PPI use reduces the risk of aspiration pneumonia in patients with reflux after resection of esophageal cancer.^9,10^ Patients with a later clinical stage had a lower LCQ-MC score; this might be because the later stages of cancer generally require a greater lymph node dissection and more preoperative neoadjuvant therapy. Lymph node dissection could lead to a higher possibility of exposing more cough receptors located in the larynx, trachea, carina, and large pulmonary bronchi.^11^ At present, we have no measures to change the two factors of clinical stage and anastomotic position, but we believe that EC patients with the above two factors should be considered as high-risk groups, requiring timely clinical attention and related clinical studies. We can try to construct a PPI medication regimen different from the conventional one for patients with cervical anastomosis after EC surgery, so as to solve the impact of reflux caused by high anastomosis on cough symptoms and improve cough symptoms by reducing the occurrence of reflux. At the same time, in future studies, we can develop risk prediction models to improve cough symptoms in patients after esophagectomy by identifying its predictors and implementing targeted and highly compliant predictive interventions.

A significant negative association was observed between the duration of surgery and the LCQ-MC score at 1 week after surgery. A longer duration of surgery implies a longer application of intubation for anesthesia. Recent studies have reported postoperative cough as a common complication after intubation.^12^ Nonintubated thoracoscopic lobectomy has been shown to reduce the incidence of postoperative cough.^13^ Therefore, for patients with EC, comprehensive evaluation and surgical plan should be performed before surgery to control the operation time and reduce the time of intubation anesthesia, which may be a feasible method to reduce postoperative cough symptoms.^14^ Clinical cohort studies can be conducted in the future to find the correlation between the operation time of EC and cough symptoms and even respiratory related complications.

In the present study, multivariate analyses of factors affecting postoperative cough showed that subcarinal lymph node dissection was an independent risk factor of postoperative cough at 1 week after surgery. This result is consistent with previous studies.^15,16^ Cough receptors are mainly located in the larynx, trachea, carina, and bronchi.^11^ With the resection of lymph nodes, more cough receptors may be exposed, thus increasing the likelihood of postoperative cough. As time progresses, the area of lymph node removal may heal and become covered with the repaired tissue, thereby decreasing the incidence of cough. This explains why lymph node removal in our study was no longer a factor that influenced the severity of coughing at 1 and 3 months post-surgery. A clinical trial conducted by Huang et al. showed that the patient group in which the space left after mediastinal lymph node resection was filled with fat tissue exhibited less cough severity than the non-fat filling group; this approach safely and effectively reduced the occurrence of postoperative cough and improved the quality of life of patients.^17^ Combined with the results of this study, we believe that this method is worthy of reference for patients after EC surgery, that is, autologous fat is applied to fill the space after subcarina lymph node dissection, improve postoperative cough symptoms, and improve patients’ quality of life. Clinical studies are needed to verify the effectiveness of its application.

This study found that postoperative cough was associated with the duration for which patients maintained a semi-reclining position. Previous studies have shown that bed-head elevation can significantly reduce regurgitation and substernal pain compared with sleeping in a supine position after esophagectomy.^18^ Reflux is an important cause of cough in postoperative EC patients. We found that a higher number of patients were able to regularly maintain a semi-reclining position in hospital than at home. This is likely because hospitals have beds with adjustable angles, while similar tools are not available in home settings. Therefore, we designed and developed a special semi-decumbent aid to help EC patients adopt an effective semi-decumbent position in postoperative home care. To verify its effectiveness, we conducted a multicenter randomized controlled study in China, which has completed clinical studies and is now entering the statistical phase.

Our research has two advantages. First, we utilized PROs to examine cough severity and incidence. The results were reported directly by the patient and helped to assess the regularity of postoperative cough. Second, the LCQ-MC method was used to evaluate postoperative cough in patients with EC; this method effectively evaluated the change trend of postoperative cough in patients with EC and provided a reference for clinical intervention measures.

Although the present study revealed clinically significant discoveries, it also has some limitations. First, because this was a single-center study, it may have inherent information bias. Second, the frequency of maintaining the semi-reclining position was subjective. In future studies, wearable devices can be considered to objectively and accurately record the frequency and time of patients assuming the semi-recumbent position to provide information for diagnosis and treatment.

## Conclusions

This study used PROs to assess trends in postoperative cough in patients who underwent esophagectomy for EC treatment. We found that the trend and risk factors of postoperative cough varied with time after surgery. Our study provides baseline data for postoperative cough management in patients with EC who are scheduled to undergo esophagectomy.
